# Incremental fuzzy C medoids clustering of time series data using dynamic time warping distance

**DOI:** 10.1371/journal.pone.0197499

**Published:** 2018-05-24

**Authors:** Yongli Liu, Jingli Chen, Shuai Wu, Zhizhong Liu, Hao Chao

**Affiliations:** School of Computer Science and Technology, Henan Polytechnic University, Jiaozuo, Henan, China; Jiangnan University, CHINA

## Abstract

Clustering time series data is of great significance since it could extract meaningful statistics and other characteristics. Especially in biomedical engineering, outstanding clustering algorithms for time series may help improve the health level of people. Considering data scale and time shifts of time series, in this paper, we introduce two incremental fuzzy clustering algorithms based on a Dynamic Time Warping (DTW) distance. For recruiting *Single*-*Pass* and *Online* patterns, our algorithms could handle large-scale time series data by splitting it into a set of chunks which are processed sequentially. Besides, our algorithms select DTW to measure distance of pair-wise time series and encourage higher clustering accuracy because DTW could determine an optimal match between any two time series by stretching or compressing segments of temporal data. Our new algorithms are compared to some existing prominent incremental fuzzy clustering algorithms on 12 benchmark time series datasets. The experimental results show that the proposed approaches could yield high quality clusters and were better than all the competitors in terms of clustering accuracy.

## Introduction

Time series can reveal the objective law of development of things, and therefore they are often deeply studied in such application areas as finance, engineering, environmental science and biology.

Biomedical time series often convey a large amount of information about public health. For example, an ECG (Electrocardiography) records much information about the structure of the heart and the function of its electrical conduction system, and it can be used to measure the rate and rhythm of heartbeats, the size and position of the heart chambers, the presence of any damage to the heart's muscle cells or conduction system, the effects of cardiac drugs, and the function of implanted pacemakers [1]. From this type of data, data mining can help extract valuable rule, knowledge or structure and thus becomes a preferred analysis tool. Especially clustering, one of the most important techniques in data mining, can be explored to extract information related to biological processes and diseases and has received extensive attention.

Clustering tries to divide data objects into homogeneous groups so that objects in the same group are as similar as possible and the ones in different groups are as dissimilar as possible. So far researchers have proposed a large number of clustering algorithms[2][3]. Fuzzy clustering, allowing each object belong to more than one cluster, is thought to be more consistent with human thinking than common crisp clustering.

Fuzzy C-Means (FCM) is the most well-known fuzzy clustering algorithm, and is known as the fuzzy version of the well-known traditional K-Means clustering. Actually, in fuzzy clustering, there is an alternative popular algorithm, named Fuzzy C-Medoids (FCMdd). FCM and FCMdd both try to minimize the same objective function, and finally return a partition matrix *U* and a list of cluster centers *V*. The main difference between FCM and FCMdd just lies in the formation mechanism of *V*. FCMdd selects some of the existing true objects as cluster medoids, while FCM regards some virtual objects, which are weighted average values of objects, as cluster centers. This subtle difference causes that these two algorithms have different performance characteristics: FCMdd is more resistant to noise than FCM and can more easily generate clustering results with high precision because noisy objects will impact the centroids of FCM more easily. In this respect, FCMdd is better than FCM.

Any clustering technique mainly relies on two concepts [3]: a clustering algorithm and a similarity measure. After discussing the first concept, a clustering algorithm above, we now focus on the second concept, an optimum similarity measure, which has a significant impact on clustering results. Unfortunately, there exist so many similarity measures that it is difficult for us to select an appropriate one [4]. Lack of selection criteria forces us to often choose a similarity measure at random, even though we already know its importance.

The *Euclidean* distance is the most common choice. This measure is only applicable to small-scale and equal-length time series, which limits the scope of its application. Furthermore, in time series data, it is inevitable to exist time shifts, which is an intractable issue for *Euclidean* distance. Thus it can be seen that *Euclidean* distance is not the optimal choice for time series clustering. Therefore, in this work, we select Dynamic Time Warping (DTW) distance as the similarity measure. In time series analysis, DTW is the most well-known algorithm, which is used exclusively for measuring similarity between two temporal sequences which may vary in speed. Taking into account time shifts, this algorithm calculates an optimal match between two time series and thus can compute the similarity more accurately.

Izakian et al [5] studied DTW based fuzzy clustering for time series data, and proposed three alternatives. Their work show DTW, using stretching or compressing segments of temporal data, is a desirable choice for fuzzy clustering of time series. However, their study is still limited in large-scale data processing.

As the continuous development of science and technology, together with constantly increasing of the scale of time series data, traditional methods expose some shortcomings: (1) in many cases, time series data is so big that it cannot be loaded into memory at a time, (2) and what is more, the data may arrive continuously so that there is even no way for us to get all of the data at a time. Therefore, clustering for large-scale time series data needs an incremental algorithm, whose objective is, given a sequence of time series, to construct a set of good partitions from the data stream, using a small amount of memory and time. Hore et al [6] proposed two incremental fuzzy clustering algorithms, *Single*-*Pass* FCM(spFCM) and *Online* FCM(oFCM). These two algorithms represent two implementation strategies for incremental clustering respectively, *Single*-*Pass* strategy and *Online* strategy. In the former strategy, large data is processed chunk by chunk, and the previous chunk is represented by its centroids, which will be integrated with the newly coming chunk for the next round of clustering. In the latter strategy, each chunk is classified individually and represented by its centroids, and then all the centroids generated will be grouped once again. Many studies [7–8] have shown that both of the strategies are very effective in handling large-scale data.

Note that both spFCM and oFCM use traditional *Euclidean* distance as the distance function. When considering DTW as the distance function to group time series data, the cluster centers of FCM-type clustering algorithms cannot be calculated directly [5], which significantly increases the computational difficulty. However, FCMdd needs not to calculate cluster centers, and thus its computational process will not be affected by different distance measures. In this respect, FCMdd is also superior to FCM.

Above analysis motivates us to study incremental FCMdd clustering based on DTW distance for clustering large scale time series. In this paper, two incremental fuzzy clustering algorithms are proposed, *Single*-*Pass* FCMdd based on DTW (spFDTW) and *Online* FCMdd based on DTW (oFDTW), which implement *Single*-*Pass* strategy and *Online* strategy respectively. Both of these two algorithms employ DTW distance to measure the similarity between pair-wise time series. In this way, even if there exist time shifts between two time series, these two algorithms can easily achieve higher quality clustering results because of more accurate results of similarity calculation.

The rest of this paper is organized as follows: Section 2 reviews some techniques and algorithms related to clustering time series data. Section 3 presents our incremental clustering algorithms. Section 4 discusses the experimental results. Finally, we conclude our work.

## Literature review

In this section, we will review some well-known techniques that are sufficiently relevant to our algorithms introduced in the next section, such as incremental clustering, fuzzy clustering and DTW distance et al.

### Incremental clustering

As mentioned above, there are two implementation strategies for incremental clustering, *Single*-*Pass* strategy and *Online* strategy. In either case, large data has to be divided into a set of chunks. In *Single*-*Pass* strategy, a clustering algorithm is implemented on each chunk in turn. As virtual objects, centroids of previous chunk are integrated with true objects of the newly coming chunk for the next round of clustering. Inevitably, centroids are much more important than common objects, and therefore should be assigned higher weights. The *Online* strategy includes two clustering steps. In the first step, each chunk is classified individually and represented by its centroids. In the second step, these centroids are assigned different weights and classified again.

Honda et al [7] extended traditional incremental algorithms into fuzzy co-clustering of co-occurrence matrices, and applied *Single*-*Pass* or *Online* approaches into such fuzzy clustering algorithms as categorical multivariate data (FCCM) and fuzzy CoDoK. To handle large datasets which cannot fit into memory entirely, Mei et al [8] proposed two incremental clustering algorithms. One method is a modification of the existing FCM-based incremental clustering, while the other is incremental clustering, i.e., *Single*-*Pass* or *Online*, with weighted fuzzy co-clustering. In 2016, we proposed two incremental algorithms based on information bottleneck, *Single*-*Pass* fuzzy c-means (spFCM-IB) and *Online* fuzzy c-means (oFCM-IB) [9], which modifies conventional algorithms by considering different weights for each centroid and object and scoring mutual information loss to measure the distance between centroids and objects.

Nowadays, with the increase of data size, incremental clustering has become one of the most prevalent research topics in data mining. To tackle large-scale data, in both *Single*-*Pass* and *Online* strategies, a weighted clustering algorithm is necessary. It can assign different weights to common objects and centroids since their importance and influence power are different.

### FCMdd and weighted FCMdd

Before introducing fuzzy clustering, we list the explanations on the mathematical notations used in this paper in Table 1.

**Table 1:** Dataset details.

Notation	Description
***C*,*N***	Numbers of clusters, objects
***u***_***ci***_	Fuzzy object partitioning membership
***v***_***c***_	Centroid/Medoid of the *c*-th cluster
***m***	FCM user-defined parameters
***w***	Weights of centroids/medoids and objects

As mentioned above, FCMdd is one of the representative algorithms of fuzzy clustering. The objective function minimized by this algorithm is as follows:

$$J_{FCMdd}=\sum_{c=1}^{C}\sum_{i=1}^{N}u_{ci}^{m}d(x_{i},v_{c})$$ (1)

where *x*_*i*_ is the *i*-th object, *d*(*x*_*i*_, *v*_*c*_) is the *Euclidean* distance between *x*_*i*_ and *v*_*c*_, and *m* (*m*≥1) is the fuzzifier parameter.

In incremental clustering, FCMdd has to become a weighted algorithm, which analyzes weighted datasets containing medoids and common objects with different significance. The objective function of weighted FCMdd (WFCMdd) to be minimized is as follows:

$$J_{WFCMdd}=\sum_{c=1}^{C}\sum_{i=1}^{N}w_{i}u_{ci}^{m}d(x_{i},v_{c})$$ (2)

where *w*_*i*_ is a positive real value, associating with each object *x*_*i*_. Under the constraint condition $\sum_{c=1}^{C}u_{ci}=1$, the value of *u*_*ci*_ can be calculated as follows:

$$u_{ci}={\left[\sum_{l=1}^{C}{\left(\frac{d(x_{i},v_{c})}{d(x_{i},v_{l})}\right)}^{\frac{1}{m-1}}\right]}^{-1}$$ (3)

In FCMdd, it is crucial to select the optimal object as the medoid. The common approach is to pick out the object that minimizes its distance with all objects in the datasets depending on their membership to the cluster [10]. However, the time complexity is high. Nasraoui et al [11] proposed a linearization algorithm, which only considers the *q* points that maximize the membership to each cluster as medoid candidates. Thus the medoid *v*_*c*_ of the cluster *c* is defined as follows:

$$v_{c}=\min_{x\in \xi }\sum_{i=1}^{N}w_{i}u_{ci}^{m}d(x_{i},x)$$ (4)

where *ξ* is the set of *q* medoid candidates.

Different from normal objects, medoids will be assigned higher weights in FCMdd because they usually preserve much more information. This weighted algorithm is widely used in the incremental clustering algorithm and will help improve performance of our algorithm in this paper.

### Fuzzy clustering based on DTW distance

It is a popular topic to cluster time series data. Till now, many researchers have proposed a large variety of algorithms. Li et al. [12] proposed a novel discord discovery algorithm based on bit representation clustering. After segmenting time series firstly, their algorithm merged several patterns with similar variation behaviors into a common cluster. Wang et al. [13] proposed a new clustering algorithm named weighted spherical 1-mean with phase shift (PS-WS1M), which introduced a phase adjustment procedure into the iterative clustering process. Besides these clustering algorithms mentioned above, there are also some algorithms about different distance metrics. Driemel and Sohler [14] studied the problem of clustering time series under the Fréchet distance. Xu and Wunsch [15] also discussed proximity measure in their work. Wang et al. [16] found that existing soft subspace clustering algorithms often utilized only one distance function to evaluate the distance among data items on each feature, which cannot deal with datasets with complex inner structures. Therefore, they constructed a composite kernel space and proposed a novel framework of soft subspace clustering by integrating distance metric learning in the CKS.

**Fig 1:**
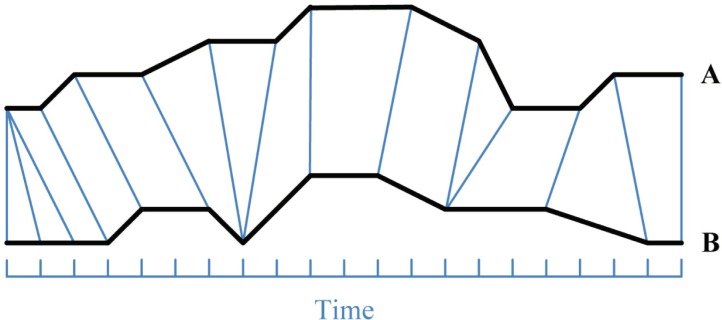
A warping between two time series.

Although there are many different distance metrics, it is known to all that DTW is a desirable choice for measuring similarity between two temporal sequences which may vary in speed. By stretching or shrinking time series along the time axis, DTW can find the optimal alignment between two time series. Fig 1 illustrates the principle of DTW. In Fig 1, for example, there are two time series, A and B, and each vertical line connects a point in A to its correspondingly similar point in B. The irregular distribution of these vertical lines shows that time series may be “warped” non-linearly by stretching or shrinking. Therefore, even if one time series may be faster than the other, or if there were accelerations and decelerations, the similarity between them could be calculated using DTW.

In general, DTW is widely studied in the fields of video, audio etc. The most representative application of DTW is automatic speech recognition, to cope with different speaking speeds. Mansour et al [17] built a system for voice recognition using dynamic time wrapping algorithm, by comparing the voice signal of the speaker with pre-stored voice signals in the database. Lee et al [18] proposed a refined DTW by adjusting the warping paths with judicially injected weights, and subsequent experiments indicate that their method significantly enhances the recognition rate compared with the DTW and HMM (Hidden Markov Model) based algorithms, especially under limited data samples.

Indeed, with the exception of video and audio data, any data, which can be turned into a linear sequence, can be analyzed with DTW. Most commonly, a time series is a sequence of discrete-time data, and thus DTW could naturally be used to analyze time series data. Guan et al [19] applied a DTW distance-based similarity measure approach and used the entire yearly NDVI(Normalized Difference Vegetation Index) time series to reduce the inaccuracy of classification using a single image. Shah et al [20] proposed to use DTW as a distance measure, rather than the *Euclidean* distance, in the framework of Learning Time-Series Shapelets for time series classification, and their extensive experimentation demonstrates statistically significant improvement in terms of wins and ranks against 13 baselines over 28 time-series datasets. These two methods were designed for time series classification. Different from classification, clustering technique, in particular fuzzy clustering, is an unsupervised learning technique, and thus has attracted more attention of researchers.

Łuczak [21] focused on a hierarchical clustering of univariate (one-dimensional) time series data, and constructed a new parametric distance function by combining DTW with Derivative Dynamic Time Warping. The experimental results demonstrated the effectiveness of the proposed approach for hierarchical clustering of time series data. For time series with cloud noise and time distortion, Zhang et al [22] proposed an effective time series clustering framework including similarity measure, prototype calculation, clustering algorithm and cloud noise handling. The core of this framework was DTW distance and its corresponding averaging method, DTW barycenter averaging (DBA). The experimental results showed that this framework performed better than classic clustering based on ordinary *Euclidean* methods. The work of Izakian et al’s [5] employed clustering techniques like FCM and FCMdd along with the DTW distance, and exploited the advantages of both the FCM and FCMdd when clustering time series. Afterwards Izakian [23] proposed an automated technique for clustering trajectory data using a Particle Swarm Optimization (PSO) approach, based on DTW distance, and the experimental results showed that the technique was able to find (near) optimal number of clusters as well as (near) optimal cluster centers during the clustering process.

## DTW based incremental fuzzy C medoids clustering

In this paper, we present two incremental FCMdd clustering algorithms based on DTW distance for clustering time series, spFDTW and oFDTW. The significant difference between spFDTW and oFDTW lies in the way in which the centroids of each chunk are handled. Similarly to some incremental clustering algorithms [9], the large-scale time series data will be split into a set of chunks, and each chunk has its own number of objects. In our work, let us suppose there are *M* chunks in total, which are available in turn.

In our spFDTW and oFDTW, a weighted fuzzy clustering algorithm based on DTW (WFCMdd-DTW) is necessary, which is similar to FCMdd. The main difference between WFCMdd-DTW and FCMdd is that WFCMdd-DTW uses DTW as similarity measure.

### WFCMdd-DTW

The objective function of WFCMdd-DTW is:

$$J_{WFCMdd-DTW}=\sum_{c=1}^{C}\sum_{i=1}^{N}w_{i}u_{ci}^{m}dtw(x_{i},v_{c})$$ (5)

where the function *dtw*(*x*_*i*_, *v*_*c*_) is the DTW distance between time series *x*_*i*_ and the medoid *v*_*c*_.

Given two time series ***a*** and ***b***, with length *S* and *T* respectively, the value of *dtw*(***a***, ***b***) is calculated using the DTW algorithm. In this algorithm, each point in ***a*** is compared with any point in ***b***. As a result, the similar shapelets from ***a*** and ***b*** will be found, although they may occur in different time periods. The pseudo-code [24] for calculating DTW distance between ***a*** and ***b*** is detailed as follows.

**Algorithm: DTW algorithm**

1. Input: ***a*** = <*a*_1_,…,*a*_*S*_>, ***b*** = <*b*_1_,…,*b*_*T*_>

2. Output:

3.     ***cost***: a matrix of size *S*×*T* containing the cost values. *cost*(*S*, *T*) is the DTW distance between ***a*** and ***b***

4.     ***path***: a matrix of size *S*×*T* containing a warping path

5. Method:

6.     Let *δ* be a distance between coordinates of sequences

7.     *cost*(1,1) = *δ*(*a*_1_,*b*_1_);

8.     *path*(1,1) = (0,0);

9.     **for** *i* = 2,3,…*S* **do**

10.         *cost*(*i*,1) = *cost*(*i*-1,1)+*δ*(*a*_*i*_,*b*_1_);

11.     **end for**

12.     **for** *j* = 2,3,…*T* **do**

13.         *cost*(1,*j*) = *cost*(1,*j*-1)+*δ*(*a*_1_,*b*_*j*_);

14.     **end for**

15.     **for** *i* = 2,3,…*S* **do**

16.         **for** *j* = 2,3,…*T* **do**

17.             *cost*(*i*,*j*) = *min*(*cost*(*i*-1,*j*), *cost*(*i*,*j*-1), *cost*(*i*-1,*j*-1))+*δ*(*a*_*i*_,*b*_*j*_);

18.             *path*(*i*,*j*) = *min_index*((*i*-1,*j*), (*i*,*j*-1), (*i*-1,*j*-1));

19.         **end for**

20.     **end for**

According to the process of WFCMdd, we can get the values of *u*_*ci*_ and *v*_*c*_ as:

$$u_{ci}={\left[\sum_{l=1}^{C}{\left(\frac{dtw(x_{i},v_{c})}{dtw(x_{i},v_{l})}\right)}^{\frac{1}{m-1}}\right]}^{-1}$$ (6)

$$v_{c}=\arg\;\min_{x}\sum_{i=1}^{N}w_{i}u_{ci}^{m}dtw(x_{i},x)$$ (7)

The solution of the constrained optimization problem in Eq (5) can be approximated by Picard iteration through Eqs (6) and (7).

### spFDTW and oFDTW

The spFDTW and oFDTW are both designed by iteratively applying the WFCMdd-DTW clustering algorithm on data chunks. In this section, we detail these two algorithms respectively.

The spFDTW is a *Single*-*Pass* incremental algorithm. In this algorithm, we implement WFCMdd-DTW on the previous chunk and generate the corresponding medoids. Compared with common objects, these medoids are obviously much more important. We therefore assign higher weights to these medoids, merge them with the common objects of the next chunk and carry out WFCMdd-DTW once again.

In spFDTW, the weight *w*_*c*_ for a medoid *v*_*c*_ of the *p*-th chunk is calculated as follows:

$$w_{c}=\sum_{i=1}^{|p|+r}u_{ci}w_{i}$$ (8)

where |*p*| is the number of objects in the *p*-th chunk, and *r* is the number of previous medoids that are added into current chunk. The value of *r* is calculated as,

$$r=\left\{ \begin{array}{ll} 0, & p=1\\ C, & p>1 \end{array}\right.$$ (9)

When we are processing the first chunk (*p* = 1), the value of *r* is 0, and the weight of each time series equals 1. After carrying out WFCMdd-DTW on this chunk, we get *C* clusters. Each cluster is represented by its medoid, whose weight is calculated using Eq (8). Now we complete clustering the first chunk, and merge the *C* medoids generated from Chunk 1 with common time series in Chunk 2. It should be noted that the weight of each common time series in Chunk 2 all equals 1, which shows that the medoids are more important. We implement WFCMdd-DTW once again on those objects including the *C* medoids generated from Chunk 1 and common time series in Chunk 2, and get *C* new clusters and new medoids, which will be merged into the Chunk 3. Repeat this procedure until the last chunk is processed, and the spFDTW terminates.

The spFDTW is outlined as follows.

**Algorithm: spFDTW algorithm**

1. Input: *C*, *p*, |*p*|, *m*

2. Output: fuzzy partitioning membership

3. Method:

4.     **for** *p* from the first to the last chunk **do**

5.         **if** (*p* = = 1) **then**

6.             perform WFCMdd-DTW on the first chunk;

7.             calculate weights of the *C* medoids;

8.         **else**

9.             perform WFCMdd-DTW on *C* medoids of previous chunk and |*p*| objects of this chunk;

10.             calculate weights of the new *C* medoids;

11.         **end if**

12.     **end for**

13.     re-calculate fuzzy memberships for all time series;

Different from spFDTW, oFDTW could be seen as a parallel clustering algorithm. The parallelism reflects the treatment of chunks. In oFDTW, WFCMdd-DTW is performed on each chunk individually. When the medoids of all the chunks are obtained, WFCMdd-DTW is implemented on all these medoids once again. The weight *w*_*c*_ for each centroid *v*_*c*_ of the *p*-th chunk is calculated as follows.

$$w_{c}=\sum_{i=1}^{\left|p\right|}u_{ci}w_{i}$$ (10)

The oFDTW is outlined as follows.

**Algorithm: oFDTW algorithm**

1. Input: *C*, *p*, |*p*|, *m*

2. Output: fuzzy partitioning membership

3. Method:

4.     **for** *p* from the first to the last chunk **do**

5.         perform WFCMdd-DTW on the *p*-th chunk;

6.         add the *C* medoids of current chunk to the centroid set;

7.         calculate weights of the *C* medoids;

8.     **end for**

9.     perform WFCMdd-DTW on the centroid set;

10.    re-calculate fuzzy memberships for all time series;

As mentioned above, our algorithms are implemented by iteratively applying the WFCMdd-DTW clustering algorithm on data chunks. Therefore, the complexities of spFDTW and oFDTW depends on the complexity of WFCMdd-DTW.

Time complexity of the WFCMdd algorithm is *O*(C*N*^2^*τ*) [25], where *τ* is the iteration number. In this paper, we extend WFCMdd into WFCMdd-DTW. And the distance measure accordingly becomes from *Euclidean* distance to DTW distance. If we calculate two time series with length *K*, the time complexity of *Euclidean* distance is *O*(*K*), while the complexity of DTW is *O*(*K*^2^). Therefore, we conclude that the complexity of WFCMdd(with *Euclidean* distance) is *O*(*CKN*^2^), and the complexity of WFCMdd-DTW(with DTW distance) is *O*(*CK*^2^*N*^2^). The spFDTW and oFDTW have the same time complexity with *O*(*CK*^2^*N*^2^*τM*). It is obvious that adoption of DTW further improve the computational complexity. However, because our work in this paper concentrates on clustering accuracy, the computational complexity is not brought into sharp focus. Otherwise, such updated versions of DTW as FastDTW [26] and SparseDTW [27], whose complexity is also *O*(*K*) and the same to *Euclidean* distance, should be more highly esteemed. And we think it will be one of our potential research directions in future.

## Experiments

To verify the performances of our algorithms, we carried out abundant experiments. In our experiments, we compared our algorithms with four incremental clustering algorithms: spFCM, spFCMdd, oFCM, oFCMdd, and a FCMdd algorithm based on DTW, named FCMddDTW[5].

### Datasets

In our experiments, we select 12 benchmark datasets from the UCR Time Series Classification Archive [28]. Among the 12 datasets, eight ones are directly related to biomedical engineering, which can examine clustering performance in the field of biology, and four ones are common datasets, which can verify the generality of clustering algorithms. These datasets are detailed in Table 2.

**Table 2:** Dataset details.

Dataset	Length	Samples	Classes	Brief
**Trace(TR)**	275	100	4	It is a synthetic dataset designed to simulate instrumentation failures in a nuclear power plant.
**Symbols(SY)**	398	995	6	Thirteen people participated in this experiment. They were asked to copy the randomly appearing symbol as best they could. There were 3 possible symbols, each person contributed about 30 attempts. The data is the X-Axis motion in drawing the shape.
**ECG5000(ECG)**	140	4500	5	5,000 heartbeats randomly chosen from a 20-hour long electrocardiogram(ECG) dataset from Physionet.
**ECGFiveDays(ECGFD)**	136	861	2	Data is from a 67 year old male. The two classes are simply ECG date.
**TwoLeadECG(TLE)**	82	1139	2	TwoLeadECG is an ECG dataset taken from physionet by Eamonn Keogh.
**ProximalPhalanxTW(PPTW)**	80	400	6	A part of Luke Davis's PhD titled "Predictive Modelling of Bone Ageing".
**DistalPhalanxOutlineAgeGroup(DPOAG)**	80	400	3	A part of Luke Davis's PhD titled "Predictive Modelling of Bone Ageing".
**PhalangesOutlinesCorrect(POC)**	80	858	2	A part of Luke Davis's PhD titled "Predictive Modelling of Bone Ageing".
**ProximalPhalanxOutlineCorrect(PPOC)**	80	291	2	A part of Luke Davis's PhD titled "Predictive Modelling of Bone Ageing".
**DistalPhalanxOutlineCorrect(DPOC)**	80	600	2	A part of Luke Davis's PhD titled "Predictive Modelling of Bone Ageing".
**Wafer (WF)**	152	6164	2	This dataset was formatted by R. Olszewski. Wafer data relates to semi-conductor microelectronics fabrication.
**ItalyPowerDemand(IPD)**	24	1029	2	It was derived from twelve monthly electrical power demand time series from Italy.

### Evaluation criteria

After grouping time series data, we need to validate the quality of final clustering results. There are numerous evaluation measures to validate the clustering quality, such as Entropy, F-Measure and Purity. In this paper, we select four evaluation criteria, F-Measure, Entropy, p-value and Clustering Score(CS).

F-Measure is the weighted harmonic mean of precision and recall. Given cluster *j* and class *i*, the values of *Precision* and *Recall* could be calculated as follows.

$$recall(i,j)=n_{ij}/n_{i}$$ (11)

$$precision(i,j)=n_{ij}/n_{j}$$ (12)

where *n*_*ij*_ is the number of time series of class *i* in cluster *j*, *n*_*i*_ and *n*_*j*_ are number of time series in class *i* and cluster *j* respectively. The final value of F-Measure of clustering results, *F*_*c*_, is calculated as below.

$$F_{c}=\sum_{i}\frac{n_{i}}{n}*\max\{F(i,j)\}$$ (13)

$$F(i,j)=\frac{2*precision(i,j)*recall(i,j)}{precision(i,j)+recall(i,j)}$$ (14)

Nowadays, F-Measure has often been used to evaluate clustering quality. In general, the higher the value of F-Measure, the better the clustering quality.

Entropy is an information theoretic measure, which examines how the documents in all categories are distributed within each cluster [29]. A lower entropy value depicts better cluster quality. The expression for Entropy of the whole clustering result is listed as follows:

$$E_{cs}=\sum_{j=1}^{C}\frac{n_{j}E_{j}}{N}$$ (15)

where *E*_*cs*_ is the whole Entropy value, *n*_*j*_ is the number of objects in cluster *j* and *E*_*j*_ is the Entropy value of cluster *j*, which is calculated using the following formula:

$$E_{j}=-\sum_{i}^{}p_{ij}\log p_{ij}$$ (16)

where *p*_*ij*_ is the probability that one document belonging to class *i* could be put into cluster *j* during the partition.

In statistical hypothesis testing, the p-value is the probability for a given statistical model that, when the null hypothesis is true, the statistical summary would be the same as or of greater magnitude than the actual observed results [30]. The use of p-values in statistical hypothesis testing is common in many fields of research such as economics, finance, et al. In research of GO (Gene Ontology) whose objective is to provide controlled vocabularies for the description of the biological process, molecular function, and cellular component of gene products, the p-value is often used to calculate the statistical significance of a group of proteins that share a GO term [31]. In the dataset, given *N* proteins where *M* of them have the same annotation, the probability of observing *m* or more proteins that are annotated with the same GO term out of *n* proteins is,

$$p-value=\sum_{i=m}^{n}\frac{\left(\begin{array}{c} M\\ i \end{array}\right)\left(\begin{array}{c} N-M\\ n-i \end{array}\right)}{\left(\begin{array}{c} N\\ n \end{array}\right)}$$ (17)

A cluster with a smaller p-value is usually more significant than one with a higher p-value. After getting the p-value of each single cluster, the quality of overall clusters could be measured by the CS function, which is calculated as follows.

$$CS=\frac{\sum_{i=1}^{ns}min\left(p_{i}\right)+\left(nl*cutoff\right)}{ns+nl}$$ (18)

where *ns* and *nl* are the number of significant and insignificant clusters, respectively. The *cutoff* denotes the *α* level (0.05), and if a group of proteins are associated with a p-value less than the cutoff, they are considered significant, and vice versa. The *min*(*p*_*i*_) is the smallest p-value of the significant cluster *i*.

### Experimental setting

In our experiments, the value of *m* is set to 2.3. For spFDTW, oFDTW, spFCMdd and oFCMdd, the termination condition is that the medoids obtained are the same to the medoids of previous iteration. It should be noted that we give the convergence analysis in Appendix A of our work. For both the spFCM and oFCM algorithms, when the number of iterations is above 50 or |*U*_*iter*+1_-*U*_*iter*_|<0.0001, the clustering process terminates, where *U*_*iter*_ stands for the partition matrix in iteration *iter*.

Here we discuss how to choose the initial medoids for our algorithms. There are many methods for choosing initial medoids. A common approach is to randomly pick several objects as medoids. This approach has the advantages of simplicity and quick run speed. However, it is not appropriate for WFCMdd-DTW, the core of our algorithms, because WFCMdd-DTW is a little sensitive to initial medoids. In our experiments, we select the following approach to initialize our algorithms. First the initial approach tries to find the first medoid which minimizes the sum of its distance with all other objects. Next the initial approach finds the object which has the longest distance to the first medoid as the second medoid. Then we determine the third medoid which maximizes the sum of its distance with previous two medoids. According to above steps, we can find all the initial medoids that are relatively far away from each other.

It is necessary to note that, since the initial medoids are not randomly picked, clustering results of such algorithms as FCMddDTW, spFDTW, oFDTW, spFCMdd and oFCMdd, will be constant, and therefore their standard deviation in terms of F-Measure and Entropy will be 0 (as Table 3 and Table 4).

**Table 3:** Comparison of the *Single*-*Pass* fuzzy clustering algorithms in terms of F-Measure and Entropy. The bold values highlight the best results obtained in each case.

Datasets	F-Measure				Entropy			
	spFCM	spFCMdd	FCMddDTW	spFDTW	spFCM	spFCMdd	FCMddDTW	spFDTW
***TR***	0.5692(0.0028)	0.6466(0)	0.7820(0)	**0.8410(0)**	0.2950(0.0020)	0.2920(0)	0.1373(0)	**0.1281(0)**
***SY***	0.7683(0.0000)	0.8802(0)	0.8332(0)	**0.9057(0)**	0.1902(0.0000)	0.1683(0)	**0.1271(0)**	0.1484(0)
***ECG***	0.7068(0.0132)	0.7505(0)	0.5874(0)	**0.7838(0)**	0.2373(0.0071)	0.2206(0)	**0.1785(0)**	0.1904(0)
***ECGFD***	0.5221(0.0000)	0.5644(0)	0.5116(0)	**0.6244(0)**	0.3006(0.0000)	0.3003(0)	0.3010(0)	**0.2983(0)**
***TLE***	0.5475(0.0000)	0.5752(0)	0.5303(0)	**0.7225(0)**	0.2999(0.0000)	0.2995(0)	0.3001(0)	**0.2530(0)**
***PPTW***	0.6336(0.0351)	0.5795(0)	0.5286(0)	**0.6661(0)**	0.2740(0.0014)	**0.2474(0)**	0.2595(0)	0.2487(0)
***DPOAG***	0.7245(0.0357)	0.7942(0)	0.7776(0)	**0.8040(0)**	0.2342(0.0038)	0.2386(0)	0.1941(0)	**0.2237(0)**
***POC***	0.5156(0.0000)	0.5179(0)	**0.6818(0)**	0.6276(0)	0.2898(0.0000)	0.2898(0)	0.2894(0)	**0.2803(0)**
***PPOC***	0.6063(0.0016)	0.5742(0)	0.6513(0)	**0.7036(0)**	0.2621(0.0002)	0.2646(0)	0.2338(0)	**0.2513(0)**
***DPOC***	0.5336(0.0000)	0.6693(0)	0.5784(0)	**0.6825(0)**	0.2856(0.0000)	0.2802(0)	0.2827(0)	**0.2794(0)**
***WF***	0.6986(0.0000)	**0.6989(0)**	0.6985(0)	0.6983(0)	**0.1485(0.0000)**	**0.1485(0)**	0.1486(0)	0.1486(0)
***IPD***	0.6123(0.0069)	**0.6318(0)**	0.6195(0)	0.6086(0)	**0.2950(0.0021)**	0.2953(0)	0.2988(0)	0.3010(0)

**Table 4:** Comparison of the *Online* fuzzy clustering algorithms in terms of F-Measure and Entropy. The bold values highlight the best results obtained in each case.

Datasets	F-Measure				Entropy			
	oFCM	oFCMdd	FCMddDTW	oFDTW	oFCM	oFCMdd	FCMddDTW	oFDTW
***TR***	0.5634(0.0028)	0.6375(0)	0.7820(0)	**0.8199(0)**	0.2949(0.0012)	0.3130(0)	0.1373(0)	**0.1540(0)**
***SY***	0.8049(0.0029)	0.8151(0)	**0.8332(0)**	0.7747(0)	0.1721(0.0027)	0.1652(0)	**0.1271(0)**	0.1662(0)
***ECG***	0.5852(0.0074)	**0.8084(0)**	0.5874(0)	0.7518(0)	0.2325(0.0509)	**0.1423(0)**	0.1785(0)	0.1489(0)
***ECGFD***	0.5154(0.0000)	0.5099(0)	0.5116(0)	**0.6224(0)**	0.3008(0.0000)	0.3009(0)	0.3010(0)	**0.2989(0)**
***TLE***	0.5334(0.0005)	0.5310(0)	0.5303(0)	**0.6621(0)**	0.3000(0.0000)	0.3001(0)	0.3001(0)	**0.2975(0)**
***PPTW***	0.5642(0.0203)	**0.7406(0)**	0.5286(0)	0.6325(0)	0.2501(0.0222)	0.2397(0)	0.2595(0)	**0.2373(0)**
***DPOAG***	0.6926(0.0003)	0.7934(0)	0.7776(0)	**0.8604(0)**	0.3244(0.0000)	0.2297(0)	0.1941(0)	**0.1664(0)**
***POC***	0.6297(0.0000)	**0.6818(0)**	0.6818(0)	**0.6818(0)**	**0.2789(0.0000)**	0.2894(0)	0.2894(0)	0.2894(0)
***PPOC***	0.6042(0.0000)	**0.6580(0)**	0.6513(0)	0.6482(0)	0.2419(0.0000)	**0.2305(0)**	0.2338(0)	0.2361(0)
***DPOC***	0.5905(0.0000)	**0.6867(0)**	0.5784(0)	**0.6867(0)**	0.2861(0.0000)	**0.2855(0)**	0.2827(0)	**0.2855(0)**
***WF***	**0.6989(0.0000)**	0.6988(0)	0.6985(0)	0.6983(0)	**0.1485(0.0000)**	**0.1485(0)**	0.1486(0)	0.1486(0)
***IPD***	0.6168(0.0046)	**0.6278(0)**	0.6195(0)	0.6229(0)	0.2995(0.0005)	**0.2982(0)**	0.2988(0)	0.2997(0)

### Experimental results

**Fig 2:**
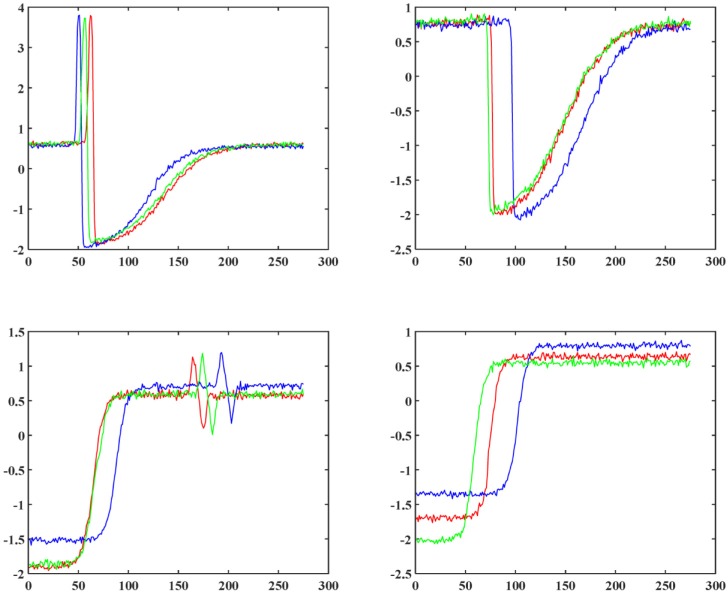
Random example time series from 4 classes of the TR dataset.

**Fig 3:**
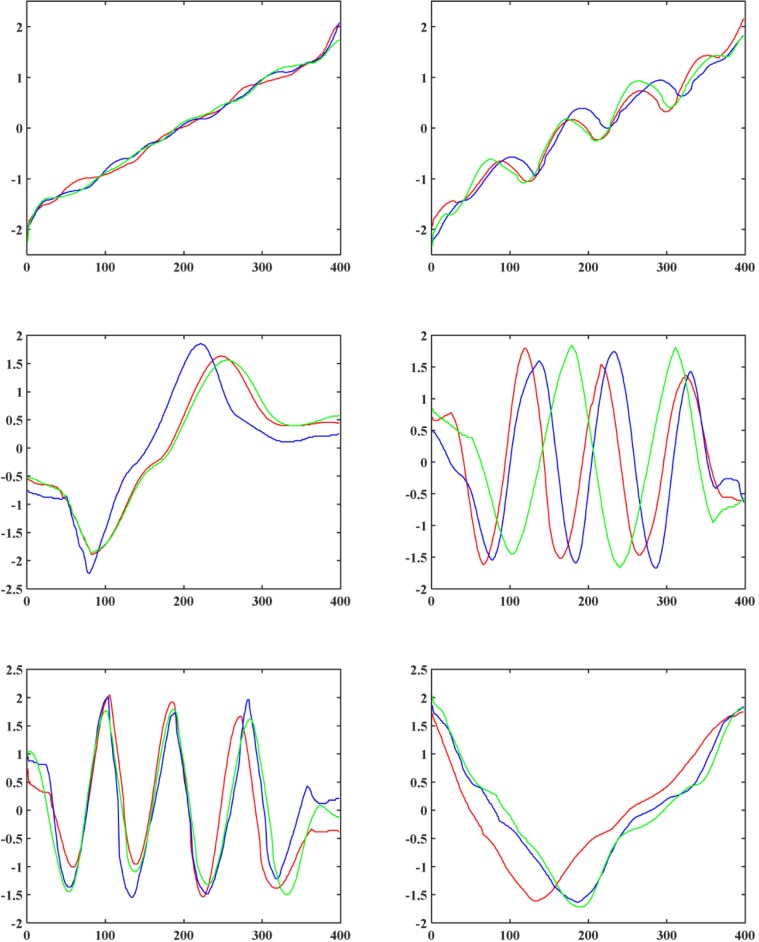
Random example time series from 6 classes of the SY dataset.

In order to evaluate the performances of clustering algorithms intuitively, we plot medoids of two datasets, TR and SY (The first two datasets in Table 2), with 4 and 6 classes respectively. In Fig 2 and Fig 3, we select randomly and plot three time series from each class of the TR and SY datasets respectively.

**Fig 4:**
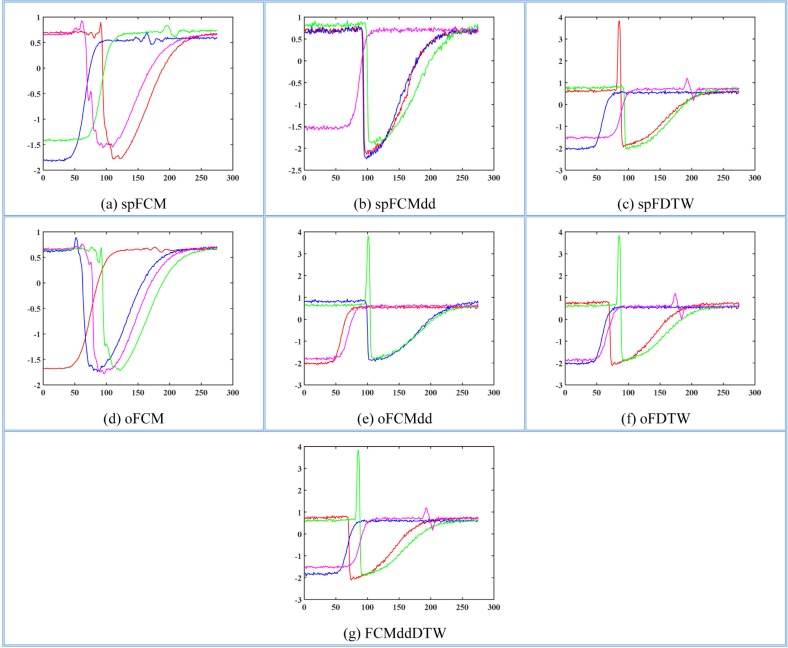
Medoids of the TR dataset using (a) spFCM, (b) spFCMdd, (c) spFDTW, (d) oFCM, (e) oFCMdd, (f) oFDTW and (g) FCMddDTW.

**Fig 5:**
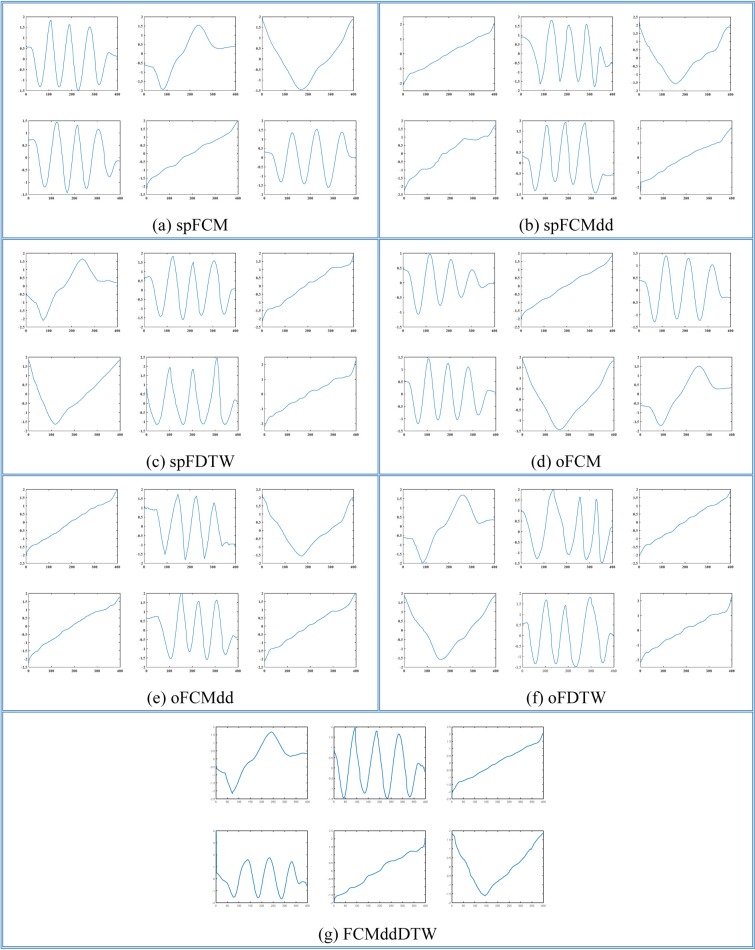
Medoids of the SY dataset using (a) spFCM, (b) spFCMdd, (c) spFDTW, (d) oFCM, (e) oFCMdd, (f) oFDTW and (g) FCMddDTW.

After observing the shapes of example time series, we carried out such clustering algorithms as spFCM, spFCMdd, spFDTW, oFCM, oFCMdd, oFDTW and FCMddDTW on the 12 datasets. In like manner, we graphically represent medoids of these two datasets, TR and SY, as Fig 4 and Fig 5 respectively. On TR, the number of clusters was set to 4. And on SY, the number of clusters was set to 6. Let us consider Fig 2 and Fig 4, which show benchmark medoids and obtained medoids of the TR dataset respectively. In clustering results of spFCM and oFCM as shown in Fig 4A and Fig 4D, only 2 classes are revealed. The fact is there are 4 benchmark classes on the TR dataset, which shows that both spFCM and oFCM lose 2 classes. The clustering results of spFCMdd (Fig 4B) are better, and this algorithm discovers 3 classes. Other algorithms, including FCMddDTW, oFCMdd, spFDTW and oFDTW, reveal all the 4 classes marked by the TR dataset. Now let us consider the SY dataset, whose benchmark medoids and obtained medoids are shown as Fig 3 and Fig 5 respectively. Because there are more benchmark classes in this dataset, it is cluttered to plot all the classes in a single graph. And thus, in Fig 5, we provide each cluster with a separate graph. In addition, it should be noted that some benchmark classes are very similar. For example, in Fig 3, the example time series of the first and second classes are similar, and so are time series of the fourth and fifth classes. It greatly increases the difficulty of clustering. As shown in Fig 5, both spFCM and oFCM reveal 5 classes. Although time series of the fourth and fifth classes in Fig 3 are very similar, these two algorithms find the two classes precisely. The spFCMdd discovers 4 classes, and the oFCMdd finds 5 classes.

These two algorithms reveal some similar classes, however both lose one class (the third class in Fig 3), whose shape is obviously different from other classes. The FCMddDTW and spFDTW discover all the 6 classes of the SY dataset, and therefore have the best clustering results. The quality of oFDTW is slightly less than spFDTW, because the oFDTW works barely satisfactory in distinguishing similar classes.

After feeling the clustering results intuitively, we have to quantify clustering results in order to compare correctly clustering performance of these clustering algorithms.

Table 3 illustrates comparison of spFCM, spFCMdd, FCMddDTW and spFDTW, in terms of F-Measure and Entropy (with the value of standard deviation). As shown in Table 3, spFDTW achieves the highest F-Measure values on all the 9 datasets, and the lowest Entropy values on 7 datasets. The average F-Measure values of these four algorithms are 0.62, 0.66, 0.65 and 0.72 respectively, and the average Entropy values are 0.26, 0.25, 0.23 and 0.23 respectively. Even on POC, WF and IPD, the three datasets where the spFDTW cannot perform the best in terms of F-Measure, the F-Measure value is near-optimal. In brief, this set of experimental results show that the spFDTW is better than or comparable to spFCM, spFCMdd and FCMddDTW in terms of F-Measure and Entropy.

**Fig 6:**
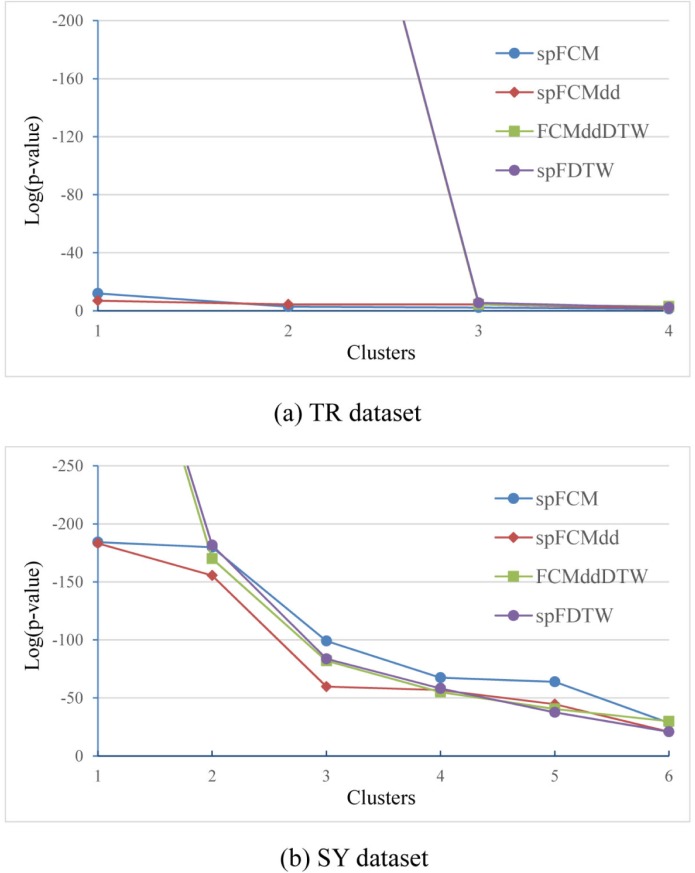
Comparison of the *Single*-*Pass* fuzzy clustering algorithms in terms of p-value on two datasets, (a) TR and (b) SY.

To further compare clustering quality of these three *Single*-*Pass* algorithms, we calculated p-values and CS based on clustering results. Confined to the length of this paper, we just give the p-value results on TR and SY, as Fig 6A and Fig 6B respectively. In Fig 6A, spFCM, spFCMdd, FCMddDTW and spFDTW all split the TR dataset into four clusters, which are sorted according to the p-values calculated. In other words, each Cluster 1, having the lowest p-value, is the most important and accurate cluster of the corresponding algorithm. Fig 6B shows the SY dataset in exactly the same way, and the difference lies in the number of final clusters. It can be seen from Fig 6 that our spFDTW has the Cluster 1 with the lowest p-value, and other clusters with comparable p-values. Results of this set of experiments show that data can be grouped into more meaningful clusters, and our algorithm could provide more significant clusters.

**Fig 7:**
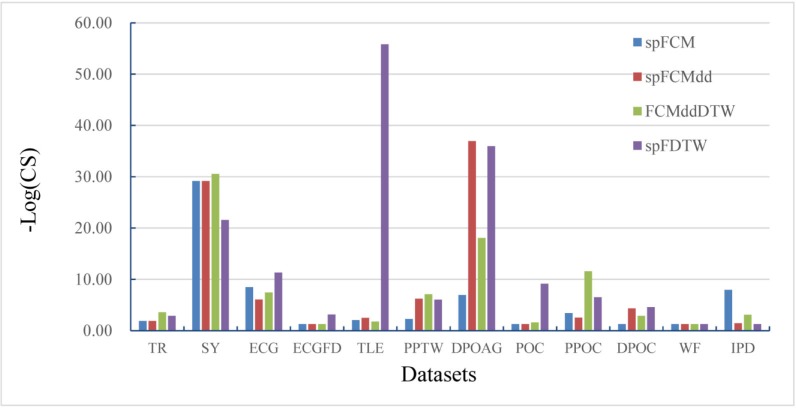
Comparison of the *Single*-*Pass* fuzzy clustering algorithms in terms of -Log_10_(CS).

Fig 7 shows the comparison of three *Single*-*Pass* approaches on the 12 datasets in terms of -Log_10_(CS). The average -Log_10_(CS) values of these four algorithms are 5.63, 7.93, 7.53 and 13.31 respectively, which shows clustering score values of our spFDTW are much lower, and thus this algorithm achieves a significant improvement than spFCM, spHFCM and FCMddDTW.

After comparing the three *Single*-*Pass* approaches, we begin to analyze experimental results of the three *Online* algorithms, oFCM, oFCMdd and oFDTW. In Table 4, these three algorithms and the FCMddDTW are compared, in terms of F-Measure and Entropy. As shown in Table 4, oFDTW achieves the highest F-Measure values on 6 datasets, and the lowest Entropy values on 6 datasets. There are 6 datasets where the oFDTW cannot perform the best in terms of F-Measure, SY, ECG, PPTW, PPOC, WF and IPD. On four of these six datasets, the oFCMdd achieves the highest clustering accuracy, and the FCMddDTW and oFDTW earns the second and third best accuracy. In terms of Entropy, although there are six datasets where the oFDTW cannot perform the best, SY, ECG, POC, PPOC, WF and IPD, the Entropy values of oFDTW are all near-optimal. The average F-Measure values of these four (oFCM, oFCMdd, FCMddDTW, oFDTW) algorithms on the twelve datasets are 0.62, 0.68, 0.650 and 0.71 respectively, and the average Entropy values are 0.26, 0.25, 0.23 and 0.23 respectively. It shows that the oFDTW is better than other three algorithms in terms of F-Measure and Entropy on these datasets.

Another thing to highlight from Table 3 and Table 4 is the comparison between the *Single*-*Pass* mode and the *Online* mode. As shown in Table 3 and Table 4, the average F-Measure values of spFDTW and oFDTW are 0.72 and 0.71 respectively, and the average Entropy values are both 0.23, which shows the spFDTW is comparable to oFDTW in terms of accuracy. On some datasets, spFDTW are better. And oFDTW can also exhibit better performance on some datasets.

The comparison between the *Euclidean* distance and DTW distance can also be provided based on Tables 3 and 4. Table 3 illustrates the results of spFCM, spFCMdd, FCMddDTW and spFDTW. Among these four algorithms, spFCM and spFCMdd use the *Euclidean* distance, and FCMddDTW and spFDTW employ the DTW distance. The results in Table 3 show that our spFDTW is the best, FCMddDTW is better than spFCM and slightly worse than spFCMdd. It tells us that, although FCMddDTW uses the DTW distance that is more suitable for time series data, its clustering accuracy does not make a significant improvement. Both spFCM and spFCMdd use traditional *Euclidean* distance which is considered to be outdated, however their accuracy is comparable because they employ an incremental mode which assigns different weights to objects according to their different importance. So we can see that although DTW is considered more accurate than *Euclidean* distance in time series data analysis, it might be not decisive in incremental clustering. Besides the distance measure, the weighted clustering algorithm is also important. The similar conclusion can also be drawn from the experimental results of oFCM, oFCMdd, FCMddDTW and oFDTW as Table 4. In a word, in incremental clustering, not only distance measure but also weighted algorithm are both important. Therefore, the improvements of our spFDTW and oFDTW come from not only the DTW distance but also the incremental clustering mechanism.

**Fig 8:**
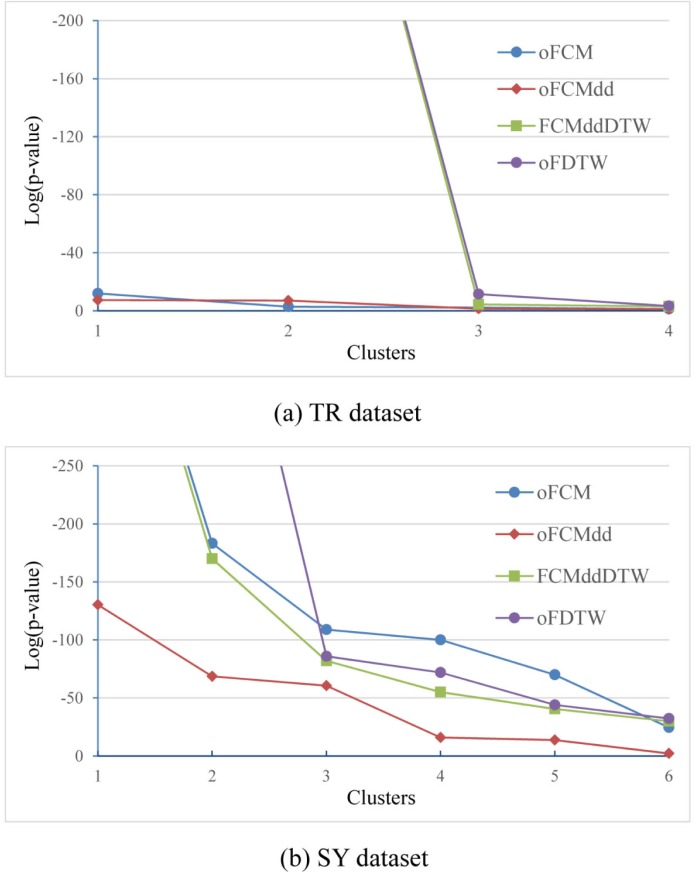
Comparison of the *Online* fuzzy clustering algorithms in terms of p-value on two datasets, (a) TR and (b) SY.

Now we continue to study the experimental results of *Online* algorithms. Like above work of the *Single*-*Pass* mode, we calculated p-values and CS based on clustering results for the three *Online* algorithms and FCMddDTW. Similarly, confined to the length of this paper, we just give the p-value results on TR and SY, as Fig 8A and Fig 8B respectively. In Fig 8A, four clusters, generated by oFCM, oFCMdd, FCMddDTW and oFDTW algorithms on the TR dataset, are sorted according to the p-values calculated. Fig 8B shows the clustering results in terms of p-values on SY dataset. It can be seen from Fig 8 that our oFDTW has the Cluster 1 with the lowest p-value, and other clusters with comparable p-values. Results of this set of experiments show that our oFDTW could also provide more significant clusters.

**Fig 9:**
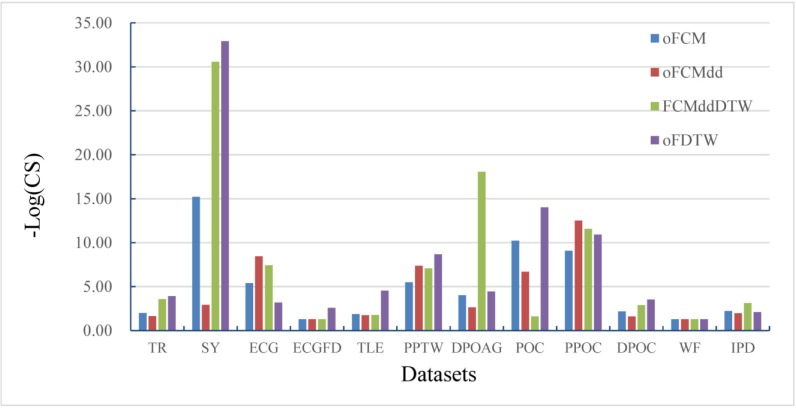
Comparison of the *Online* fuzzy clustering algorithms in terms of -Log_10_(CS).

Fig 9 shows the comparison of three *Online* approaches and FCMddDTW on the 12 datasets in terms of–Log_10_(CS). The average -Log_10_(CS) values of these four algorithms are 5.03, 4.19, 7.53 and 7.69 respectively, which shows clustering score values of our oFDTW are much lower, and thus this algorithm achieves a significant improvement than other three algorithms.

## Conclusion

Most commonly, a time series is a series of data points listed in time order. Time series data often contains the natural laws of things, which invests time series analysis great importance. Nowadays, we usually use such techniques as data mining for analyzing time series data in order to extract meaningful statistics and other characteristics of the data.

With the scale of time series data constantly expanding, in order to group time series data, researchers designed some incremental clustering algorithms, such as spFCMdd and oFCMdd. However, as we all know, for calculating the pair-wise similarity of time series, employing a DTW distance is a desirable choice. Therefore, in this paper, we propose two incremental clustering algorithms, spFDTW and oFDTW. Coincident with employing the DTW similarity measure, these two algorithms select FCMdd as the kernel, instead of FCM, which could mitigate the impact of noisy data and help to improve clustering accuracy. In order to verify the effectiveness of our algorithms, we carried out experiments on twelve datasets including general datasets and biomedical datasets, and experimental results show that our algorithms outperform some existing prominent *Single*-*Pass* and *Online* fuzzy clustering algorithms.

## Appendix A

In this section, we will study the convergence of spFDTW and oFDTW. Since these two algorithms both have WFCMdd-DTW as the basic algorithm, we need only to prove the convergence of WFCMdd-DTW.

It is known to all that a monotone bounded function is convergent. There are therefore two theorems to be proven. The first theorem is that the value of *J*_*WFCMdd*-*DTW*_ in Eq (5) never increases in the process of WFCMdd-DTW, and the second theorem is that *J*_*WFCMdd-DTW*_ is a bounded function.

### Theorem 1

In every iteration of WFCMdd-DTW, the newer value of *u*_*ci*_ never increases the value of the objective function *J*_*WFCMdd*-*DTW*_ in Eq (5).

### Proof

In the objective function *J*_*WFCMdd-DTW*_ in Eq (5), if we consider the parameter *u*_*ci*_ as a single variable and other parameters as constants, the function will be rewritten as:

$$J_{WFCMdd-DTW}(U)=\sum_{c=1}^{C}\sum_{i=1}^{N}w_{i}u_{ci}^{m}dtw(x_{i},v_{c})$$ (19)

Now, Theorem 1 can be proven by showing that the u^*^ (the updated *u*_*ci*_ given by Eq (6)) is the local minima of the objective function *J*_*WFCMdd*-*DTW*_(*U*) by Lagrange multiplier method. Let us consider the following Hessian matrix Δ^2^*J*_*WFCMdd*-*DTW*_(u^*^) first.

$$\begin{array}{l} \Delta^{2}J_{WFCMdd-DTW}(u)=\left[\begin{array}{ccc} \frac{\partial^{2}J(u)}{\partial u_{11}\partial u_{11}} & \cdots & \frac{\partial^{2}J(u)}{\partial u_{11}\partial u_{CN}}\\ \vdots & \ddots & \vdots \\ \frac{\partial^{2}J(u)}{\partial u_{CN}\partial u_{11}} & \cdots & \frac{\partial^{2}J(u)}{\partial u_{CN}\partial u_{CN}} \end{array}\right]\\ \;=\left[\begin{array}{ccc} w_{1}m(m-1)dtw(x_{1},v_{1})u_{11}^{m-2} & \cdots & 0\\ \vdots & \ddots & \vdots \\ 0 & \cdots & w_{N}m(m-1)dtw(x_{N},v_{c})u_{CN}^{m-2} \end{array}\right] \end{array}$$ (20)

It is obvious that the Hessian matrixΔ^2^*J*(u^*^) is positive definite. Moreover, before we deduce the value of *u*_*ci*_ as Eq (6), we let (∂*J*_*WFCMdd*-*DTW*_(*u*_*ci*_)/∂*u*_*ci*_) = 0. Therefore, the updated *u*_*ci*_ is indeed a local minima of *J*_*WFCMdd*_, and it never increases the objective function value.

### Theorem 2

In every iteration of WFCMdd-DTW, the newer value of *v*_*c*_ never increases the value of the objective function *J*_*WFCMdd*-*DTW*_ in Eq (5).

### Proof

Following the method used by Carvalho et al. [25], we prove this theorem in this section. We consider the parameter *v*_*c*_ in Eq (5) as a single variable and other parameters as constants. Let *t* be the iteration number. The objective function *J*_*WFCMdd-DTW*_ in Eq (5) will be rewritten as:

$$J_{WFCMdd-DTW}(V)=\sum_{c=1}^{C}\sum_{i=1}^{N}w_{i}u_{ci}^{m}dtw(x_{i},v_{c})$$ (21)

Now, we need to prove that *J*_*WFCMdd-DTW*_(*vt c*)≥*J*_*WFCMdd-DTW*_(*vt*+1 *c*), which means that the value of *J*_*WFCMdd-DTW*_(*V*) does not increase at each iteration.

In order to search for the best cluster medoid, the WFCMdd-DTW algorithm starts from (*V*^(*t*-1)^,*W*^(*t*-1)^,*U*^(*t*-1)^) and ends with (*V*^(*t*)^,*W*^(*t*-1)^,*U*^(*t*-1)^), *t* is the number of iteration, moreover, *W*^(*t*-1)^ = (*w*_1_^(*t*-1)^,*…*,*w*_*c*_^(*t*-1)^) and *U*^(*t*-1)^ = (*u*_1_^(*t*-1)^,*…*,*u*_*n*_^(*t*-1)^), which represent the *w*_*i*_ and *u*_*ci*_ in Eq (5) respectively, are kept fixed while the cluster medoid *v*_*c*_ in Eq (5) is updated. Thus, we can rewrite the objective function *J*_*WFCMdd-DTW*_ in Eq (5) as:

$$J(V^{\left(t\right)})=J(v_{1}^{\left(t\right)},\ldots ,v_{c}^{\left(t\right)})=\sum_{c=1}^{C}J_{c}(v_{c}^{\left(t\right)})=\sum_{c=1}^{C}\sum_{i=1}^{N}{\left(u_{ci}^{\left(t-1\right)}\right)}^{m}w_{c}^{\left(t-1\right)}dtw(x_{i},v_{c}^{\left(t\right)})$$ (22)

Because

$$J(V^{\left(t\right)},W^{\left(t\right)},U^{\left(t\right)})=\sum_{c=1}^{C}\sum_{i=1}^{N}w_{c}^{\left(t\right)}{\left(u_{ci}^{\left(t\right)}\right)}^{m}dtw(x_{i},v_{c}^{\left(t\right)})$$ (23)

and

$$J(V^{\left(t+1\right)},W^{\left(t\right)},U^{\left(t\right)})=\sum_{c=1}^{C}\sum_{i=1}^{N}w_{c}^{\left(t\right)}{\left(u_{ci}^{\left(t\right)}\right)}^{m}dtw(x_{i},v_{c}^{\left(t+1\right)})$$ (24)

Then we have,

$$V^{\left(t+1\right)}=\underset{V=(v_{1},\ldots v_{c})}{\arg\;\min}\sum_{c=1}^{C}\;\sum_{i=1}^{N}w_{c}^{\left(t\right)}{\left(u_{ci}^{\left(t\right)}\right)}^{m}dtw(x_{i},v_{c}^{\left(t+1\right)})$$ (25)

and thus,

$$J\left(V^{\left(t\right)},W^{\left(t\right)},U^{\left(t\right)}\right)⩾J\left(V^{\left(t+1\right)},W^{\left(t\right)},U^{\left(t\right)}\right).$$ (26)

Therefore Theorem 2 holds.

### Theorem 3

In every iteration of WFCMdd-DTW, the newer value of *w*_*i*_ never increases the value of the objective function *J*_*WFCMdd-DTW*_ in Eq (5) and does not change its convergence property.

### Proof

In order to compute the weight parameter, the WFCMdd-DTW starts with (*V*^(*t*-1)^,*W*^(*t*-1)^,*U*^(*t*-1)^) and ends with (*V*^(*t*-1)^,*W*^(*t*)^,*U*^(*t*-1)^), moreover, *V*^(*t*-1)^ = (*v*_1_^(*t*-1)^,…,*v*_*c*_^(*t*-1)^) and *U*^(*t*-1)^ = (*u*_1_^(*t*-1)^,…,*u*_*n*_^(*t*-1)^), which represent the *v*_*c*_ and *u*_*ci*_ in Eq (5) respectively, are kept fixed while the weight parameter is updated. Thus, we can rewrite the objective function *J*_*WFCMdd-DTW*_ in Eq (5) as

$$\begin{array}{l} J(W^{\left(t\right)})=J(w_{1}^{\left(t\right)},\ldots ,w_{c}^{\left(t\right)})=\sum_{c=1}^{C}J_{c}(w_{c}^{\left(t\right)})=\sum_{c=1}^{C}\sum_{j=1}^{P}w_{c}^{\left(t\right)}J_{c}\\ =\sum_{c=1}^{C}\sum_{i=1}^{N}{\left(u_{ci}^{\left(t-1\right)}\right)}^{m}w_{c}^{\left(t\right)}dtw(x_{i},v_{c}^{\left(t-1\right)}) \end{array}$$ (27)

Because

$$J(V^{\left(t\right)},W^{\left(t\right)},U^{\left(t\right)})=\sum_{c=1}^{C}\sum_{i=1}^{N}w_{c}^{\left(t\right)}{\left(u_{ci}^{\left(t\right)}\right)}^{m}dtw(x_{i},v_{c}^{\left(t\right)})$$ (28)

and

$$J(V^{\left(t\right)},W^{\left(t+1\right)},U^{\left(t\right)})=\sum_{c=1}^{C}\sum_{i=1}^{N}w_{c}^{\left(t+1\right)}{\left(u_{ci}^{\left(t\right)}\right)}^{m}dtw(x_{i},v_{c}^{\left(t\right)})$$ (29)

Then we have,

$$W^{\left(t+1\right)}=(w_{1}^{\left(t+1\right)},\ldots ,w_{c}^{\left(t+1\right)})=\underset{W=(w_{1},\ldots ,w_{c})}{\arg\;\min}\sum_{c=1}^{C}\;\sum_{i=1}^{N}w_{c}^{\left(t+1\right)}{\left(u_{ci}^{\left(t\right)}\right)}^{m}dtw(x_{i},v_{c}^{\left(t\right)})$$ (30)

and thus,

$$J\left(V^{\left(t\right)},W^{\left(t\right)},U^{\left(t\right)}\right)⩾J\left(V^{\left(t\right)},W^{\left(t+1\right)},U^{\left(t\right)}\right).$$ (31)

Assume that the stationarity of the *J*_*WFCMdd-DTW*_ is achieved in the iteration *t* = *T*. Then we have that *J*_(*T*)_ *= J*_(*T*+1)_ and then *J*(*V*^(*t*)^,*W*^(*t*)^,*U*^(*t*)^) = *J*(*V*^(*t*+1)^,*W*^(*t*+1)^,*U*^(*t*+1)^). When the membership degree represented by *U*^(*T*)^ and the weight parameter *W*^(*T*)^ which are the same to *u*_*ci*_ and *w*_*i*_ in Eq (5) separately are kept fixed, we can conclude that,

$$J\left(V^{\left(t\right)},W^{\left(t\right)},U^{\left(t\right)}\right)=J\left(V^{\left(t+1\right)},W^{\left(t\right)},U^{\left(t\right)}\right).$$ (32)

When the membership degree represented by *U*^(*T*)^ and the medoid *V*^(*T*)^ which are the same to *u*_*ci*_ and *v*_*c*_ in Eq (5) separately are kept fixed, we can conclude that,

$$J\left(V^{\left(t+1\right)},W^{\left(t\right)},U^{\left(t\right)}\right)=J\left(V^{\left(t+1\right)},W^{\left(t+1\right)},U^{\left(t\right)}\right).$$ (33)

Thus, we have,

$$J\left(V^{\left(t\right)},W^{\left(t\right)},U^{\left(t\right)}\right)=J\left(V^{\left(t+1\right)},W^{\left(t\right)},U^{\left(t\right)}\right)=J\left(V^{\left(t+1\right)},W^{\left(t+1\right)},U^{\left(t\right)}\right).$$ (34)

Therefore the Theorem 3 holds for all *t*≥*T*.

### Theorem 4

The objective function of *J*_*WFCMdd-DTW*_ in Eq (5) is bounded.

### Proof

Since the minimum value of *u*_*ci*_ is 0, and *dtw*(*x*_*i*_, *v*_*c*_)≥0, we know that the value of *J*_*WFCMdd-DTW*_ is greater than or equal to 0. In other words, this function is bounded.

### Corollary 1

The WFCMdd-DTW algorithm converges to a local minimum of the optimization, with the update formulae above.

### Proof

Theorems 2–3 indicate that the procedure of membership updating never increases the value of *J*_*WFCMdd-DTW*_. And Theorem 4 tells us the objective function of *J*_*WFCMdd-DTW*_ is bounded. Therefore, the iteration process should stop somewhere before or when it reaches the limit.
